# Spectrum of Celiac disease in Paediatric population: Experience of Tertiary Care Center from Pakistan

**DOI:** 10.12669/pjms.336.13489

**Published:** 2017

**Authors:** Danish Abdul Aziz, Misha Kahlid, Fozia Memon, Kamran Sadiq

**Affiliations:** 1 Dr. Danish Abdul Aziz, MBBS, MRCPCH, FCPS. Senior Instructor, Department of Paediatrics, Aga Khan University Hospital, Karachi, Pakistan; 2 Misha Khalid, Final Year of MBBS Medical Student, Aga Khan University Hospital, Karachi, Pakistan; 3 Dr. Fozia Memon, MBBS, FCPS, Chief Resident, Department of Paediatrics, Aga Khan University Hospital, Karachi, Pakistan; 4 Dr. Kamran Sadiq, MBBS, FCPS. Assistant Professor and Paediatric Gastroenterologist, Department of Paediatrics, Aga Khan University Hospital, Karachi, Pakistan

**Keywords:** Celiac Disease, Gluten free Diet, Chronic Diarrhea, Serum Tissue Transglutaminase antibodies

## Abstract

**Objective:**

To determine clinical features and relevant laboratory investigations of patient with celiac disease (CD) and comparing classical celiac disease (CCD) with Non-diarrheal celiac disease (NDCD).

**Methods:**

This is a five years retrospective study conducted at The Aga Khan University Hospital Karachi, Pakistan from January 2010 to December 2015, enrolling children from one year to 15 years of either gender diagnosed as celiac disease in accordance with revised ESPGHAN criteria. Biopsy samples with grade 2 or more on Modified Marsh Classification were considered as consistent with celiac disease. Celiac patients were categorized into Classical celiac disease (with Chronic Diarrhea) and non-diarrheal celiac disease (Atypical celiac) and their clinical features and relevant laboratory investigations were documented.

**Results:**

Total 66 patients were selected with celiac disease according to inclusion criteria, 39 (59.09%) patients were labeled as CCD and 27 (40.91%) patients were labeled as NDCD. Marsh grading 3a and above were more marked in CCD as compared to NDCD. Mean titer for Tissue transglutaminase antibodies (TTG) were higher in CCD group in comparison to NDCD group. In CCD, the most common clinical presentations were abdominal distension whereas in NDCD, the most remarkable features were recurrent abdominal pain (62.9%). Frequency of failure to thrive is significantly high in CCD (82.05%) but patients merely with short stature were more common in NDCD (33.3%). Refractory anemia was present in 66.6% patients in NDCD group and 41.1% patients in CCD group. 74.3% patients in CCD group were vitamin D deficient whereas 85% patient had vitamin D deficiency in NDCD group (p= 0.03).

**Conclusion:**

NDCD is not uncommon in our population. Recurrent abdominal pain, failure to thrive or patients only with short stature and refractory anemia are prominent features in NCDC group whereas abdominal distension, failure to thrive and recurrent abdominal pain were noticeable features in CCD. High grade histopathology and raised antibodies titer is hallmark of CCD. Vitamin D deficiency is almost equally present in both groups.

## INTRODCTION

Celiac disease is a permanent intolerance to gluten due to chronic systemic autoimmune process in genetically susceptible individuals resulting in injury to small intestine mucosa.^1^ The prevalence of Celiac disease is about 1% around the globe with female to male ratio of 2:1 to 3:1.^2^

Children with celiac disease usually present with chronic diarrhea, failure to thrive, abdominal distension, abdominal pain, vomiting and anorexia^3^. Clinical symptoms and initial presentations divide the CD into diarrheal or classical CD (CCD) and non-diarrheal or atypical CD (NDCD). Classical CD presents with chronic diarrhea as chief complaints along with some other related symptoms. In absence of typical diarrheal presentation, many cases with CD are ignored and NDCD remains an underdiagnosed entity in our region.^4^ Non-diarrheal CD (NDCD) has variable presentation including failure to thrive, short stature, refractory anemia, rickets, vomiting and recurrent oral ulcers.^5^-^9^ Identifying these features early in disease and investigating promptly with serology and upper GI endoscopy and biopsy will facilitate early diagnosis and timely application of gluten free diet. This further improves the overall quality of life of such children and decreases the risk of future complications and malignancies.^10^

Comparing clinical presentations, laboratory investigations and co-morbid conditions of CCD and NDCD will help us to understand the different spectrum of celiac disease. Aim of this study was to highlight these clinical and laboratory features identified in our population.

## METHODS

This is 5 years retrospective study conducted at Aga khan University Hospital Karachi, Pakistan from January 2010 to December 2015 enrolling children from 1 year to 15 years of either gender diagnosed as celiac disease (CD) in accordance with revised ESPGHAN criteria.^11^ All children on gluten diet with raised anti-tissue transglutaminase titer (>18 IU/L) went through upper GI endoscopy and duodenal biopsies. Modified Marsh Classification was used to grade the histopathology of biopsy samples. Biopsy samples with grade 2 or more were considered as consistent with diagnosis of celiac disease. Chronic Diarrhea was defined as passage of watery or semi-solid stools or increased liquidity in stool consistency as reported by the attended/ child for more than 14 days. Patients who did not present with chronic diarrhea were labeled as “Non-diarrheal celiac patients”. Therefore, celiac patients were categorized into Classical (with Chronic Diarrhea) and non-diarrheal celiac disease (Atypical celiac) and their clinical features and relevant laboratory investigations were documented. Both groups were started on gluten free diet and micronutrient supplements and their symptomatic improvement was documented after three months on gluten free diet. Markers of improvement include return of normal stool consistency with less liquidity (in CCD), improvement in weight (crossing up at least one centile line), and increase in hemoglobin level ≥2 g/dl (both in CCD and NDCD). Short stature was defined as height for age less than -2 Z-score (height or length less than two standard deviations for age and gender). Failure to thrive was identified as growth (weight for age) below 3^rd^ centile or weight deceleration that crosses two major percentile lines on a growth chart. Patients with persistent anemia (hemoglobin<10g/dl) not responding to three months of dietary modification, iron and folate supplementations and after excluding common hemoglobinopathies were labeled as having refractory anemia. Rickets was defined on clinical examination according to age with patients showing frontal bossing, widely open anterior fontanel, widening of the wrist joint, and bowing of the legs. Radiographic examination of wrist joint further helped in diagnosis rickets showing cupping, fraying and splaying of wrist joint. Intestinal and extra-intestinal clinical presentations were documented for both groups of celiac disease. Pertinent investigations including hemoglobin level, vitamin D level, transaminases and serum albumin were noted. Charts were reviewed for certain comorbid conditions such as autoimmune thyroiditis, insulin dependent diabetes mellitus, Down’s syndrome, epilepsy and IgA deficiency for two categories of CD. Data from annual screening tests was retrieved for all patients for thyroid profile. Serum IgA levels were done for enrolled patients at the time of antibody screening along with TTG IgA profile to rule out IgA deficiency in order to identify possible false negative screening results.

All continuous variables were analyzed with t-test and all proportions were analyzed with chi square test. SSPS v.20 was used to analyze the results.

## RESULTS

**Fig.1:**
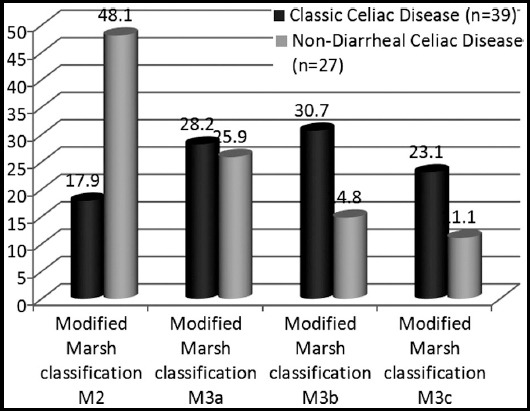
Patients distribution on Modified Marsh Classification Histopathology for Duodenal Biopsies.

Total 66 patients were selected with celiac disease according to inclusion criteria, out of these 39 (59.09%) patients had classical presentation of celiac disease with chronic diarrhea and 27 (40.91%) patients had atypical presentation without chronic diarrhea labeled to have non-diarrheal celiac disease (NDCD). Male to female ratio was 1.4. Total celiac patients enrolled in the study had age range from 16 months to 175 months. Patients with non-diarrheal celiac disease had symptoms later in life as compared to classical disease. At the same time patients with NDCD had extended duration of symptoms prior to diagnosis with mean age at diagnosis of 7.32±1.67 years in comparison with CCD where mean age at diagnosis was 5.21±1.34 years. Table-I shows demographic presentation of CCD and NDCD. Marsh grading 3a and above were more marked in CCD as compared to NDCD as shown in Fig.1.

**Table-I:** Demographic feature of celiac patients.

	*Classical (n=39) 59.09%*	*Atypical (n=27) 40.91%*	*Total (n= 66)*
Mean age at onset of symptoms (years)	3.53±1.26	4.24±1.74	3.88±1.38
Mean age at diagnosis (years)	5.21±1.34	7.32±1.67	6.25±1.56
Male	22(56.41%)	17(62.96 %)	39 (59.09%)
Female	17(43.58%)	10 (37.03%)	27(40.91%)
Male : Female Ratio (M:F)	1.29	1.70	1.44
Family History	3 (7.69%)	1 (3.70%)	4 (6.06%)
Consanguinity	14 (35.89%)	13(48.14%)	27 (40.90%)
Mean weight (Kg)	15.23±1.89	18.15±1.67	15.93±1.78
Mean Height (cm)	102.12±1.37	112.40±1.56	105.02±1.43

**Fig.2:**
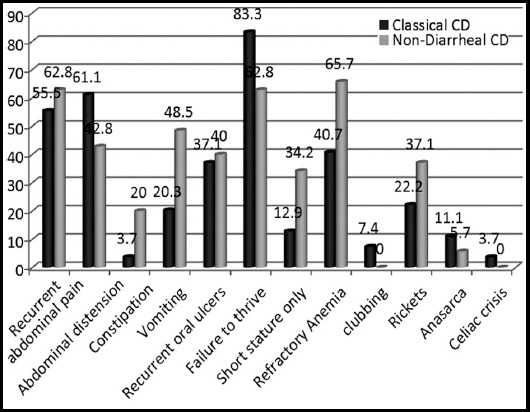
Clinical presentations in Paediatric Celiac Population.

In Classical disease, the most common clinical presentations were abdominal distension 24(61.5%) and recurrent abdominal pain 21 (53.8%). In NDCD, the most remarkable features were recurrent abdominal pain 17 (62.9%) and vomiting 13 (48.1%) followed by abdominal distension 12 (44.4%), recurrent oral ulcers 11 (40.7%) and constipation 6 (22.2%). Frequency failure to thrive is significantly high in CCD but patients only with short stature were more common in NDCD. Fig.2 shows different clinical features in two groups.

Mean titer for Tissue transglutaminase antibodies (TTG) were higher in CCD group in comparison to NDCD group (p=0.04). Vitamin D was significantly low in both group with 29 (74.3%) patients had vitamin D deficiency in CCD group and 23(85.2%) patient in NDCD group are vitamin D deficient (p= 0.03). Laboratory investigations and their comparison between two groups of celiac patients are shown in Table-II. Frequencies of different associated co-morbid conditions are mentioned in Table-III.

**Table-II:** Laboratory Investigation of Celiac Patients.

	*Classical (n=39)*	*Non-diarrheal (n=27)*	*p-value*
Mean Serum TTG IgA (IU/L)	125±54	85±34	0.04
Mean Serum TTG IgG (IU/L)	220±97	148±76	0.01
Vitamin D Deficiency (<30ng/ml)	29 (74.3%)	23(85.2%)	0.03
Mean serum vitamin D level (ng/ml)	15.5±1.7	12.4±1.4	0.05
Iron deficiency Anemia hemoglobin <10gm/dl & serum ferritin< 10ng/ml)	18 (46.1%)	19(70.3%)	0.40
Mean hemoglobin(g/dl)	9.2±1.2	6.9±2.4	0.39
Hypertransaminases (AST>35U/L, ALT>45U/L)	3(7.6%)	6 (22.2%)	0.14
Mean serum Aspartate aminotransferases (AST) U/L	27.9±1.23	34±1.55	0.30
Mean serum Alanine aminotransferase (ALT) U/L	36±1.02	43±1.32	0.36
Hypoalbuminemia (serum albumin<3gm/dL)	18 (46.1%)	7(25.9%)	0.15
Mean Serum Albumin	2.76±0.97	3.1 ±0.89	0.83

**Table-III:** Co-morbid Associated Conditions in Celiac Patients

	*Classical (n=39)*	*Non-diarrheal (n=27)*
Down Syndrome	2(5.1%)	1(3.7%)
Insulin depended diabetes mellitus (IDDM)	1(2.5%)	1(3.7%)
Dermatitis herpetiformis	1(2.5%)	0
Autoimmune Thyroiditis	1(2.5%)	1(3.7)
IgA deficiency	2(5.1%)	1(3.7%)
Epilepsy	3(7.6%)	2(7.4%)

## DISCUSSION

This tertiary care based retrospective study has shown that non-diarrheal celiac disease (NDCD) in not infrequent in our population. Around 40% patients in our study population did not have chronic diarrhea at the time of diagnosis which is quite similar to Kuloglu et al.^12^ It is evident in many series that patients with classical presentation were identified at earlier age than those with atypical presentation without diarrhea.^13^ Similar scenario has been noticed in our celiac cohort. The time duration of appearance of symptoms and diagnosis was significantly high in NDCD group in our experience. This could be partially elucidated by their varied presentation requiring high index of suspicion in comparison to CCD. Genetic basis of celiac disease is well recognized in literature and certain haplotypes like HLA -DRB1-DQA1and DQB1 are commonly associated with high risk for celiac disease. Ouda S et al. described an autosomal recessive (AR) mode of inheritance with 96%patients having parents with consanguinity in their celiac cohort^14^. In our experience, 27 (40.90%) patients had consanguineously related parents.

Marsh grade 3a and above was more commonly seen in patients with CCD in comparison with NDCD where most of the biopsies were 3a or less. This is in accordance with multiples studies which showed gastrointestinal symptoms are more common in patients with high grade small intestinal mucosahistopathology.^15^ In our population, mean serum TTGIgA and TTGIgG antibodies titers were documented to be higher in patients with CCD in comparison to NDCD. TTG IgA seropositivity is linked with more intense disease.^15^ Bhattacharya M et al. established significant correlation between TTG IgA antibodies titers and higher grades of small intestinal histopathology.^16^

In CCD group, abdominal distension was the most frequently reported complaint other than chronic diarrhea. Dinler et al. reported abdominal distension as a predominated features in patients with typical or CCD.^13^ In our experience, recurrent abdominal pain and vomiting were dominated feature in NDCD patients. Letizia et al. showed interesting relation of recurrent abdominal pain with celiac disease and found that 43% celiac patients in their population did not experience any symptoms other than recurrent abdominal pain (RAP).^17^ In our study 63% patients had recurrent abdominal pain in NDCD group and 54% patients had similar complaint in CCD group. Although recurrent abdominal pain is frequently reported with celiac disease but current evidences do not recommend screening of such children for celiac disease.^18^,^19^ In our experience, 82% patients were failure to thrive in CCD group and 63% patients in NDCD group with overall 74% patients presented with failure to thrive in our celiac cohort. This is quite similar to Aziz S et al where patients with failure to thrive were around 61%.^20^ Short stature or stunted growth can be the only presentation of celiac disease. Many studies recommend screening for celiac disease in severely stunted children.^7^,^8^,^21^ Our study has revealed that 33% patient in NDCD group merely presented with short stature.

Refractory anemia not responding to iron or folate supplement is frequently reported in celiac patients round the globe and it has been noticed that low hemoglobin may not fully improve even after a one year on a strict GFD.^22^,^23^ Our study showed 41% patient in CCD group and 66% patient in NDCD presented with refractory anemia. In our series, vitamin D deficiency was documented in 85% patient with NDCD and 74% patients with CCD. Topal et al. has shown that around 52% patients had vitamin D deficiency in celiac patients at the time of diagnosis and recommend screening for serum vitamin D levels along with zinc and iron in patients with celiac disease for prompt management.^9^ Certain co-morbid conditions and associations are linked with celiac disease including autoimmune diseases involving endocrine glands, certain syndromes, dental enamel disorders and skin diseases. In our cohort 7.5% patients have some form of epileptic disorder and were on neurology follow-up in clinics. Experience from south east Turkey has showed that the frequency of biopsy-proven celiac disease was 15.7% among children with epilepsy.^24^ In our study, 3% patients were documented to have Insulin Dependent Diabetes Mellitus (IDDM). From different experiences, the prevalence of celiac disease was noted to be 1% to 19% in patients with Type-1 diabetes mellitus and interval screening is recommended for other disease in presence of one.^25^

## CONCLUSION

NDCD is not uncommon in our population with certain important clinical presentations dominated in this group. Patients without chronic diarrhea having clinical feature of recurrent abdominal pain, vomiting, failure to thrive or merely short stature and refractory anemia should prompt physician to think and investigate for celiac disease. High grade histopathology and raised antibodies titer is the hallmark of CCD and associated with prominent gastrointestinal symptoms. Vitamin D deficiency is almost equally present in both groups. Co-morbid conditions and associations may arise during the course of the disease in these children needing timely follow-up in outpatient clinics and interval screening for certain co-morbid conditions.

### Author`s Contribution

**DAA:** Conceptualized and designed the study and drafted the initial manuscript.

**MK, FM:** Did the data collection, analysis and compiled the results.

**KS:** Helped in literature review and in editing of the manuscript for final submission.
